# Respiratory Syncytial Virus Matrix (M) Protein Interacts with Actin In Vitro and in Cell Culture

**DOI:** 10.3390/v10100535

**Published:** 2018-09-30

**Authors:** Shadi Shahriari, Ke-jun Wei, Reena Ghildyal

**Affiliations:** Centre for Research in Therapeutic Solutions, Faculty of Science and Technology, University of Canberra, Canberra ACT 2617, Australia; shadi.shahriari@canberra.edu.au (S.S.); kejunw2000@yahoo.com (K.-j.W.)

**Keywords:** actin cytoskeleton, virus transport, respiratory syncytial virus, matrix protein

## Abstract

The virus–host protein interactions that underlie respiratory syncytial virus (RSV) assembly are still not completely defined, despite almost 60 years of research. RSV buds from the apical surface of infected cells, once virion components have been transported to the budding sites. Association of RSV matrix (M) protein with the actin cytoskeleton may play a role in facilitating this transport. We have investigated the interaction of M with actin in vitro and cell culture. Purified wildtype RSV M protein was found to bind directly to polymerized actin in vitro. Vero cells were transfected to express full-length M (1–256) as a green fluorescent protein-(GFP) tagged protein, followed by treatment with the microfilament destabilizer, cytochalasin D. Destabilization of the microfilament network resulted in mislocalization of full-length M, from mostly cytoplasmic to diffused across both cytoplasm and nucleus, suggesting that M interacts with microfilaments in this system. Importantly, treatment of RSV-infected cells with cytochalasin D results in lower infectious virus titers, as well as mislocalization of M to the nucleus. Finally, using deletion mutants of M in a transfected cell system, we show that both the N- and C-terminus of the protein are required for the interaction. Together, our data suggest a possible role for M–actin interaction in transporting virion components in the infected cell.

## 1. Introduction

Respiratory syncytial virus (RSV) is a major pathogen responsible for lower respiratory tract infections (LRTI) in infants, young children, the immunosuppressed, and the elderly [1,2]. Patients with RSV infections present with clinical complications, such as pneumonia and bronchiolitis [3]. A significant challenge to RSV treatment and prevention is absence of available antiviral drugs and vaccines that effectively target infections [1,4]. RSV is an enveloped virus with a negative-sense single-stranded RNA genome, belonging to the *Pneumovirus* genus of the *Pneumoviridae* family [5,6]. The RSV genome encodes 11 proteins and is tightly encapsidated with the nucleocapsid, which is composed of the nucleocapsid (N) protein, RNA polymerase (L) and its cofactor phosphoprotein (P), as well as the M2-1 protein [5,7]. In addition to these, the genome also encodes the envelope glycoproteins fusion protein (F), glycoprotein (G) and small hydrophobic protein (SH), two non-structural proteins (NS1 and NS2), the M2-2 protein, and the matrix protein (M).

The M protein is a non-glycosylated phosphorylated protein located external to the nucleocapsid layer, where it acts as a bridge between the lipid bilayer envelope and the nucleocapsid [5,7,8,9,10,11,12]. By bridging the lipid bilayer envelope and the nucleocapsid, the M protein plays a role in driving the coordinated interplay between the viral structural components to facilitate viral assembly [13]. The RSV M is also hypothesized to mediate the transportation of newly synthesized ribonucleoprotein complexes (RNPs) to assembly sites which, in turn, drives assembly at the cell surface [5,7,13].

Another important role of the RSV M protein is its localization to the nucleus early in infection, although replication of the virus occurs in the cytoplasm [12,14]. This may be to inhibit host transcription and delay particle formation until later in infection, when there is an accumulation of viral glycoproteins and RNPs [15,16,17]. It is at this later stage in infection that the M protein associates with the RNPs to inhibit viral transcription and, ultimately, facilitate virus assembly [15].

There is an important role for the host actin cytoskeleton in the lifecycle of many viruses, including RSV [18,19]. Actin has been shown to be important in RSV infection for effective transcriptase activity in vitro [20,21]. The importance of the actin cytoskeleton in infection has been demonstrated through studies undertaken with the cytoskeleton destabilizing drug, cytochalasin D. Cytochalasin D inhibits RSV entry as well as release, reducing infectivity [5,13,22]. Cytochalasin D treatment of cells prior to RSV infection or immediately after infection leads to significant reduction in titer of released, as well as cell-associated virus, after 48 h [23].

Cellular actin is present in two forms, monomeric or polymerized actin [24]. Globular actin monomers (G-actin) polymerize (F-actin) establishing the actin filament [5,23,25]. The actin microfilament network has been suggested to play a role in the transportation of RSV components, including RNPs, to budding sites [5,26]. It has been shown that a complex containing the N, P, M2-1 and M as well as a complex containing N, P and M2-1 can bind polymerized actin. However, it is only the M-containing complex that is anchored onto the microfilament network the virus utilizes to reach budding sites at the plasma membrane [5,26,27,28]. M may be mediating RNP transport through its interactions with polymerized actin.

In this study, we have investigated the interaction of M with polymerized actin. Using in vitro assays, RSV-infected cells and cells transfected to express recombinant M protein, we show that M is indeed able to interact with polymerized actin and destabilization of microfilaments leads to relocalization of M within the cells.

## 2. Materials and Methods

### 2.1. Cells, Viruses

Human type II respiratory epithelial A549 cells and monkey kidney epithelial Cos-7 cells were obtained from the European Collection of Authenticated Cell Cultures (ECACC, Salisbury, UK) and cultured at 37 °C in 5% CO_2_. Cells were maintained in Dulbecco’s modified Eagle’s medium (DMEM) high glucose (Sigma, St. Louis, MO, USA, 6429), supplemented with 10% fetal bovine serum (FBS) (Interpath, Melbourne, VIC, Australia) and 5 mg/mL penicillin, 5 mg/mL streptomycin, and 10 mg/mL neomycin solution (PSN). RSV subtype A2 obtained from the American Type Culture Collection (ATCC, Manassas, VA, USA) was used for all virus infections and grown in Vero cells, maintained as above in DMEM with 2% FBS and PSN.

### 2.2. M Protein Expression and Purification

Codon-optimized *M* gene, cloned into the pCDF vector, was used to express M protein in *E. coli* as a fusion with 6× His, as previously published [7]. Briefly, *E. coli* DH10B cells transformed with pCDF-M were grown in the presence of spectinomycin, and expression induced with 0.5 mM IPTG for 4 h. RSV M protein was purified under native conditions using the Bio-Scale™ Mini Profinity™ IMAC Cartridges (Bio-Rad, Hercules, CA, USA), as per the manufacturer’s recommendations.

### 2.3. In Vitro Actin Binding Assay

Purified M protein eluted in the imidazole elution buffer was dialyzed overnight against the general actin buffer (G-buffer; 50 m M Tris-HCl, pH 7.5, 2 m M CaCl_2_), followed by centrifugation at 100,000× *g* for 20 min at 4 °C for use in the actin binding assay. Actin binding reactions consisted of 31.5 μg freshly prepared F-actin (or G-buffer alone) with 9 μg of 6× His-M in G-buffer with 100 mM ATP (R1441, ThermoFisher Scientific, Waltham, MA, USA). Reactions were incubated at 24 °C for 1 h before ultracentrifugation for 20 min at 100,000× *g* and 22 °C in the Hitachi CP-90WX ultracentrifuge (Hitachi Koki Co., Ltd., Hitachinaka City, Ibaraki Pref., Japan). Supernatants were transferred to new microfuge tubes and pellets were resuspended in 65 μL 1× G-buffer. Samples were then made up in Laemmli buffer and analyzed by SDS-PAGE. Samples were denatured at 95 °C for 5 min before being loaded onto 10% SDS-PAGE gel and electrophoresed. Proteins were visualized by staining with Coomassie Brilliant Blue overnight and destaining in 10% acetic acid.

### 2.4. Plasmids Used in the Study

The mammalian cell expression constructs for GFP alone, GFP-M (1–256), GFP-M (110–183), GFP-M (110–256) have been described previously [28]. The pEPI-GFP-M (1–109) expression plasmid was generated using Gateway™ Technology (Invitrogen, Carlsbad, CA, USA). The full-length M gene cloned into pDONR207 was used as a template. PCR products containing appropriate coding sequences and *attB*-flanked sites were purified using the Wizard^®^ SV Gel and PCR Clean-Up System (A9281, Promega, Madison, WI, USA), as per manufacturer’s recommendation. Entry clone pDONR207-M (1–109) was established through the Gateway^®^ BP recombinant reaction between the *attB*-flanked PCR products and entry vector pDONR207 (Life Technologies, Carlsbad, CA, USA). The gene of interest was then introduced into destination vector pEPI-DESTC using the Gateway^®^ LR recombinant reaction (Life Technologies, Carlsbad, CA, USA). The expression clone was confirmed by DNA sequencing.

### 2.5. Cytochalasin D Treatment

#### 2.5.1. Effect of Cytochalasin D on RSV Infectious Titer

Overnight, subconfluent monolayers of A549 cells in 6-well plates were infected with RSV A2 at multiplicity of infection MOI = 1. The virus inoculum was replaced with culture medium after 1 h of absorption, and this timepoint is referred to as “0” hour. Infected cells were treated with cytochalasin D (4 μg/mL) for 12 h at 6 or 18 h post-infection. The inhibitor medium was replaced with fresh medium without cytochalasin D, and cells were cultured further up to 48 h post-infection. Supernatants were collected, an equal volume of medium was added to cells, and cultures as well as supernatants were frozen at −20 °C. Frozen samples were thawed, clarified of cellular debris by centrifugation at 4500 rpm for 15 min, and the supernatant used for immunoplaque assay.

#### 2.5.2. Effect of Cytochalasin D on Localization of M in RSV-Infected Cells

Overnight subconfluent monolayers of A549 cells grown on glass coverslips were infected with RSV A2 at MOI of 1 and treated with cytochalasin D as above. Control cells received the same number of medium changes but no cytochalasin D. Cells were fixed at the indicated times with 4% formaldehyde, permeabilized with 0.5% Triton X-100, and used for immunofluorescence assay.

#### 2.5.3. Effect of Cytochalasin D on Localization of GFP-M in Transfected Cells

Overnight subconfluent monolayers of Cos-7 cells were transfected to express GFP alone, GFP-M (1–256), GFP-M (110–183), GFP-M (110–256) or GFP-M (1–109). The localization of M to the nucleus has been shown to be through the action of the 110–183 amino acid sequence, which contains the nuclear localization sequence (NLS) of the protein that allows for the targeting of GFP to the nucleus [29]. Plasmids were transfected with Lipofectamine 2000 (11668019, Invitrogen, Carlsbad, CA, USA) as per manufacturer’s recommendation. At 24 h post-transfection, cells were treated with 1 μg/mL cytochalasin D for 6 h before live cell confocal microscopy. Control cells received the same number of medium changes but no cytochalasin D.

### 2.6. Immunofluorescence Assay

Fixed, permeabilized cells on coverslips were immunostained with anti-M (gift from Erling Norrby, Sweden) mAb diluted 1:1000 in PBS. Cells were washed with PBS followed by incubation in goat anti-M-Alexa Fluor 488, and phalloidin-Alexa Fluor-594. Cells were washed, followed by 10 min incubation with 5 μL/mL Hoechst 33432 (nuclear counterstain), washed again, and coverslips were mounted with DAKO mounting media. Slides were incubated overnight in the dark to allow for maturation before confocal microscopy.

### 2.7. Confocal Microscopy

Live (transfected) or fixed (infected) cells were viewed under the 60× oil immersion objective lens on the Nikon Eclipse Ti confocal laser scanning microscope (CLSM) (Nikon Instruments Inc., Melville, NY, USA.). Images were acquired using the NIS Elements software (Version 4.0, Nikon Instruments Inc., Melville, NY, USA). To determine the relative fluorescence in the nucleus to that in the cytoplasm (Fn/c), while adjusting for background noise (Fb), the following formula was used: Fn/c = (Fn − Fb)/(Fc − Fb).

### 2.8. Immuno-Plaque Assay

Vero cells were seeded into flat-bottom 96-well plates at 1 × 10^4^ cells/well. Following overnight culture, cell monolayers were infected with serial dilutions of virus samples by absorption for 2 h before the wells were topped up with 100 μL of fresh medium. Cells were fixed with 2% H_2_O_2_ in methanol at 48 h post-infection and left to dry overnight on the bench. The plates were blocked with 1% BSA in PBS for 30 min followed by 2 h incubation with goat anti-RSV (1:1000 in 1% BSA in PBS) at room temperature. Plates were washed with phosphate buffered saline containing 0.1% Tween 20 (PBS-T) and incubated for 1 h in secondary antibody (donkey anti-goat IgG conjugated to horseradish peroxidase, diluted 1:1000 in 1% BSA in PBS). Plates were washed, and bound antibody detected by color development using diaminobenzidine chloride (DAB, Sigma, 10 mg/15 mL) in Tris-buffered saline, pH 7.5, 12 μL H_2_O_2_. Once plaques were obvious, the substrate solution was discarded, and plates dried overnight at room temperature in the dark. The following day, the now visible plaques were counted and plaque-forming units (pfu)/mL calculated.

### 2.9. ProteoExtract Subcellular Fractionation

Overnight cultures of A549 cells were infected and treated with cytochalasin D as above (Section 2.5.2), followed by subcellular fractionation at 30 h post infection. Fractionation was performed using the ProteoExtract^®^ Subcellular Proteome Extraction Kit as per manufacturer’s instructions. Four fractions were obtained including cytosol (fraction 1), membrane (fraction 2), nuclear (fraction 3), and cytoskeletal (fraction 4). Samples were then analyzed by Western blotting.

Protein samples were separated on 10% SDS-PAGE, followed by wet transfer onto nitrocellulose membrane in Tris-glycine-ethanol buffer, as described previously [30]. Membranes were incubated in blocking solution (3% low fat milk powder or 3% bovine serum albumin in PBS) for 1 h at room temperature, followed by overnight incubation in primary antibody in blocking solution at 4 °C. Membranes were probed for M protein as well as marker proteins (tubulin for cytosol, LDL-R for membrane, lamin b1 for nucleus, actin for cytoskeleton). The membranes were washed, and bound antibodies detected with Western Lightning Plus ECL kit (203–14401, PerkinElmer, Melbourne, VIC, Australia). Chemiluminescence was analyzed using the Li-COR Odyssey Fc infrared imaging system and the image was acquired using the Image Studio^TM^ software (version 5.0, Li-COR Biosciences, Lincoln, NE, USA).

The density of the M band in each fraction was quantitated, normalized to the density of M in total lysate (sum of M in all four fractions) taken as 1, and expressed as change from no treatment taken as zero.

## 3. Results

### 3.1. Microfilaments Are Important for RSV Assembly

**Figure 1:**
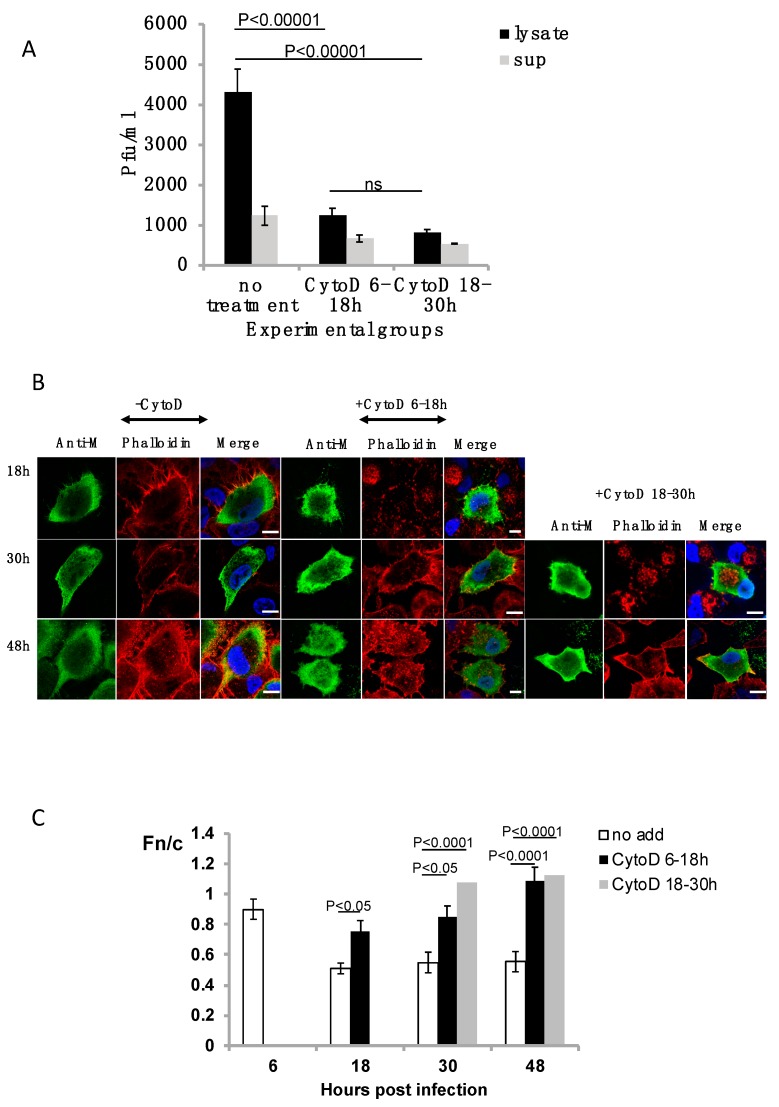
Treatment of respiratory syncytial virus (RSV)-infected A549 cells with cytochalasin D. Overnight cultures of A549 cells were infected with RSV A2 at MOI = 1. Cells were either left untreated (no treatment) or treated at early (6–18 h p.i.) or late (18–30 h p.i.) times in infection with cytochalasin D (CytoD). (**A**) A549 cells were infected with RSV (MOI = 1) and either left untreated or treated (experimental groups) with cytochalasin D early (6–18 h p.i.) or late (18–30 h p.i.) in infection. The cell lysates (lysate) and supernatants (sup) were collected at 48 h p.i., and infectious virus titer (pfu/mL) estimated by immunoplaque assay. (**B**) Infected cells were fixed at indicated times p.i. (numbers on the left) and probed for localization of M and microfilament network (phalloidin). Representative images are shown. Scale bars = 10 μm. (**C**) Images such as those shown in (**B**) were analyzed using ImageJ to determine the relative fluorescence of nucleus to cytoplasm (Fn/c) as shown. Data are mean ± SEM, *n* ≥ 15 from an experiment representative of three independent experiments. Statistically significant differences between groups are shown, ns = non-significant. *p* values were determined by 2-way ANOVA and significance accepted at *p* < 0.05.

To demonstrate that microfilaments are required for RSV assembly, we determined if viral titer is reduced upon treatment with cytochalasin D. RSV-infected cells were treated with cytochalasin D at early (6–18 h) or late (18–30 h) times in infection, and samples were collected at 48 h post-infection. Culture supernatants and cell lysates were then analyzed for infectious RSV by plaque assay. Our plaque assay results demonstrate that without treatment, the infected lysate had 4320 ± 973 plaque forming units per mL (pfu/mL) compared to the supernatant with 1233 ± 234 pfu/mL (Figure 1A). With treatment, regardless of time of treatment, the virus titer in lysates was reduced to approximately 25%. Although there was a reduction in the titer of the virus in the supernatants (released virus) on cytochalasin D treatment, this was not significantly different to the control, no treatment sample. There was no significant difference in RSV titers in supernatants or lysates between the two treatments. Our data clearly show that disruption of microfilaments with cytochalasin D leads to reduction of RSV titers, as expected.

### 3.2. Microfilaments Are Important for Appropriate Subcellular Localization of M Protein

We have previously shown that the M protein is localized in the nucleus of infected cells early in infection [12], and mostly in the cytoplasm and at the membrane later in infection. As M has a key role in RSV assembly, its presence in the cytoplasm at the appropriate time is essential for RSV assembly. We wanted to determine whether the effect of cytochalasin D on RSV titers is due to mis-localization of M in the infected cells. RSV-infected cells were either left untreated or treated with cytochalasin D at early (6–18 h) or late (18–30 h) times in infection and fixed at 18, 30, and 48 h post-infection (Figure 1B) and probed for localization of M protein (anti-M) and microfilament structure (phalloidin). As expected, M was present mostly in the cytoplasm at all times tested and the microfilament network remained intact (images labelled −CytoD). With treatment early in infection, the M protein is present in the cytoplasm, including at the cell boundary, with some M obviously localized in the nucleus (images labelled +CytoD 6–18 h). Treatment later in infection results in the localization of the M protein once again in the cytoplasm, cell boundary, and some localization to the nucleus (images labelled +CytoD 18–30 h).

Treatment with cytochalasin D, regardless of early or late treatment, resulted in the collapse of the microfilament network in infected cells (image labelled 18 h, phalloidin in the +CytoD 6–18 h column and image labelled 30 h, phalloidin in the +CytoD 18–30 h column). Interestingly, the microfilament network is seen to recover and become intact upon the removal of cytochalasin D (image labelled 30 h, phalloidin in the +CytoD 6–18 h column and image labelled 48 h, phalloidin in the +CytoD 18–30 h columnn). Analysis of the digital images, such as those in Figure 1B, confirmed these results, effectively demonstrating that the M protein has localized to the nucleus upon treatment and the consequent loss of microfilaments at 30 h post-infection (Figure 1C). There was a significant change in the Fn/c values on cytochalasin D treatment on early and late treatment. Our data strongly suggest that the microfilament network mediates, at least in part, the subcellular localization of M protein in RSV-infected cells.

**Figure 2:**
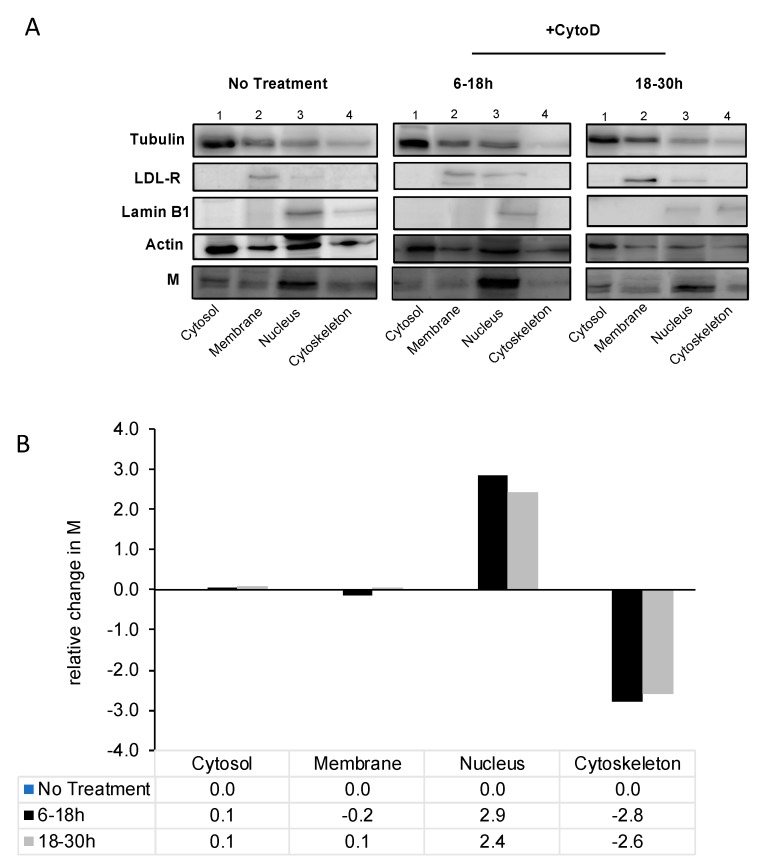
RSV M localizes to the nucleus upon microfilament destabilization. (**A**) A549 cells were cultured overnight and infected with RSV A2 at MOI = 1. Infected cells were either left untreated (−CytoD), treated (+CytoD) early in infection (6–18 h), or late in infection (18–30 h). Subcellular fractionation was undertaken to determine the presence of RSV M in cytosol, membrane, nuclear, and cytoskeletal fractions against several antibodies as controls. Controls used include anti-tubulin antibody (marker for the cytosol), anti-LDL-R (marker for membrane), anti-lamin B1 (marker for the nucleus), and anti-β-actin (marker for cytoskeleton) as shown. (**B**) Quantitative analysis of the subcellular fractionation blot as shown in (**A**) was undertaken to determine the percentage change in RSV M in each fraction after CytoD treatment, relative to no treatment.

To further confirm the nuclear localization of RSV M upon microfilament destabilization, we conducted a subcellular protein fractionation. RSV-infected cells were treated with cytochalasin D at early and late times in infection, and processed for fractionation at 48 h post-infection. Our results demonstrate changes in M localization with treatment with cytochalasin D compared to no treatment (Figure 2). M protein was observed in all four fractions in infected cells. Treatment with cytochalasin D, regardless of early or late treatment, resulted in the M protein to be increased in the nuclear fraction relative to other fractions. Image analysis confirmed this observation showing a 2.5- to 3-fold increase in M in the nucleus, relative to untreated sample, with a concomitant decrease of M in the cytoskeletal fraction. That this change in subcellular localization was specific to M is shown by the fact that there was no change in the localization of the markers for membrane (LDL-R), cytosol (tubulin) or nucleus (lamin b1) on cytochalasin D treatment compared to without treatment (e.g., compare the distribution of bands in blots labelled tubulin between “no treatment” and “+CytoD”).

Together, the data from Figure 1 and Figure 2 strongly suggest that M protein binds to polymerized actin filaments in RSV-infected cells, and that destabilization of the microfilament network leads to change in M’s localization.

### 3.3. M Protein Can Bind to Polymerized Actin In Vitro

**Figure 3:**
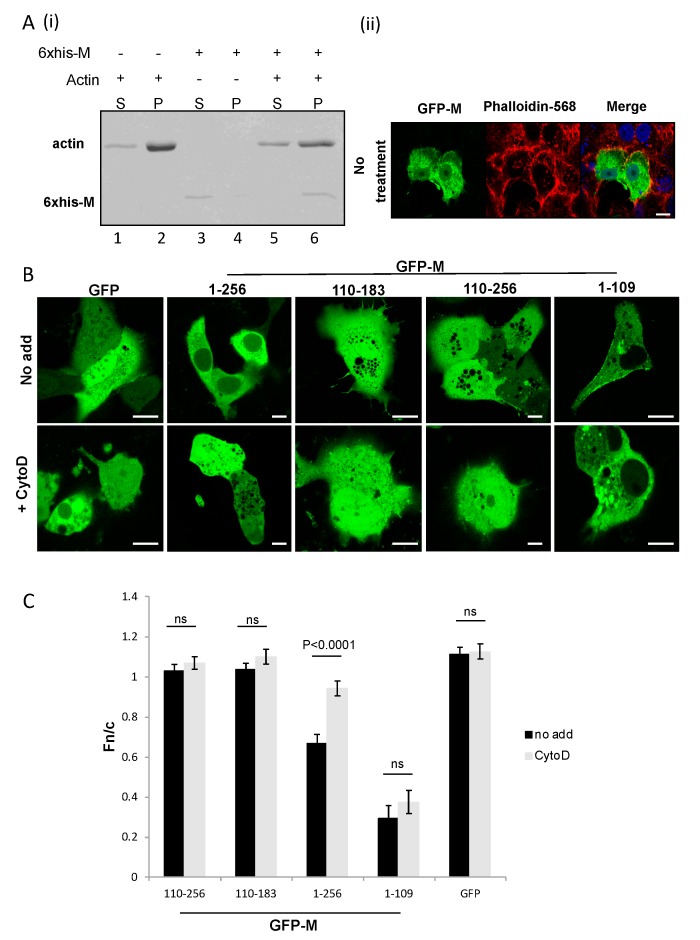
M binds polymerized actin. **A**(**i**) Recombinant purified M protein (6× His-M) was incubated with in vitro polymerized and monomeric actin, as described in the text. Samples were centrifuged to separate polymerized and monomeric actin. Supernatant (S) and pellet (P) fractions were analyzed with SDS-PAGE and visualized by Coomassie Brilliant Blue staining. The bands representing actin and RSV M are indicated. **A**(**ii**) A549 cells were cultured overnight and transfected to express full-length GFP-M (1–256). Transfected cells were left untreated and probed for visualization of the microfilament network (phalloidin-594). The colocalization of M and actin microfilaments is indicated in yellow (image labelled merge). Scale bars = 10 μm. (**B**) Cos-7 cells were cultured overnight and transfected to express GFP, GFP-M (1–256), or deletion mutants GFP-M (110–183) and GFP-M (110–256). Transfected cells were left untreated (no add) or treated with cytochalasin D (+CytoD) and imaged live using confocal microscopy. Representative images are shown. Scale bars = 10 μm. (**C**) Images such as those shown in (**B**) were analyzed by ImageJ to Fn/c. Data shown are mean ± SEM, *n* ≥ 15. Statistically significant differences between groups are shown, ns = non-significant.

We next wanted to determine if M is capable of binding directly to polymerized actin. To this end, we undertook an in vitro binding assay using recombinant purified protein and in vitro polymerized F-actin. The assay utilized lysis buffers that separate the G- (monomeric) and F- (filamentous) forms of actin by solubilizing only G-actin, while F-actin is retained in the insoluble fraction. As clearly shown in Figure 3, M is present in the supernatant (soluble fraction) in the absence of F-actin, but moves to the pellet (insoluble fraction) in its presence (compare band labelled 6× His-M in A(i) lanes 3,4 with the same band in lanes 5,6), showing that M binds polymerized actin. F-actin was present mostly in the pellet fraction, as expected (in A(i) lanes 1,2 and 5,6).

To determine if M is able to associate with microfilaments in the cellular context, we transfected Cos-7 cells with plasmid to express full-length M as a GFP fusion protein (GFP-M). Cells were fixed at 24 h post-transfection and microfilaments stained with phalloidin-594. CLSM images of the transfected cells clearly showed that M colocalized with the microfilaments at the cell periphery (Figure 3A(ii)), yellow color in the image labelled “merge”).

### 3.4. Full-Length M Protein is Responsible for Binding to Polymerized Actin

Since our results demonstrated an interaction between RSV M and actin both in cell culture and in vitro, we wanted to determine the motif within M that is required for binding to actin. Cos-7 cells were transfected to express GFP alone or full-length RSV M (1–256 aa) or its deletion mutants (110–183 aa, 110–256 aa, and 1–109 aa), with a GFP tag. Cells were either treated with cytochalasin D for 6 h or left untreated before live CLSM was undertaken at 24 h after transfection. As shown in Figure 3B, in the absence of cytochalasin D, GFP-M was mainly cytoplasmic as expected [17], becoming diffuse across both nucleus and cytoplasm on treatment (images labelled 1–256). Deletion mutants GFP-M (110–183) and GFP-M (110–256) were nuclear and cytoplasmic without treatment, as expected [17], and there was no change in their localization after treatment (images labelled 110–183 and 110–256). Deletion mutant GFP-M (1–109) was mainly cytoplasmic without treatment, as expected, as it does not contain the M NLS motif, and there was no change in its localization after treatment (images labelled 1–109). GFP was present diffused across the whole cell with or without treatment (images labelled GFP). Results were confirmed with image analysis (Figure 3C), which showed a significant change in the nuclear to cytoplasmic ration (Fn/c) of GFP-M on treatment with cytochalasin D, but no change in the localization of GFP alone, GFP-M (110–183), GFP-M (110–256), or GFP M (1–109). Our data suggest that the full 1–256 aa sequence of M is required for binding to actin. This may be due to motifs being present at more than one location that must come together to enable binding, however, this remains to be examined. Our data clearly shows that the N- or the C-terminal halves of M by themselves are not enough to enable interaction with actin filaments.

## 4. Discussion

In our study, we have shown that the RSV M protein interacts with actin in vitro and in cell culture, and that microfilaments have a role in the subcellular localization of M protein. RSV M protein has been suggested to play a role in the transportation of the RNPs to assembly sites, and the M-containing complex is suggested to be anchored onto the microfilament network that the nucleocapsid takes advantage of to reach assembly sites [24,26]. The M-actin interaction may be the process by which the RNPs of RSV are transported to plasma membrane assembly sites. Although knowledge on the involvement of actin in RSV infection is limited, actin is an internal component of purified RSV virions and is involved in the establishment of cytoplasmic extensions [24]. The formation of these structures has led to the understanding that RSV has developed an actin-based motility system for its movement for cell-to-cell spread, within the cytoplasm, and in syncytia formation [24]. Our data showing a small, non-significant change in the titer of RSV released into the cell medium on destabilization of microfilaments strongly suggests that microfilaments are involved mostly in moving components of RSV within the infected cell with limited involvement in virus release. Cytochalasin D treatment early or late in infection resulted in a similar reduction in titer suggesting that microfilaments are required throughout the whole assembly process. This is in line with a study showing that both microtubules and microfilaments have a role in RSV replication and assembly [23]. Our data are somewhat in contrast to a previous study that showed a significant reduction in released as well as cell-associated virus on cytochalasin D treatment [23], probably due to the very different experimental conditions used. Our experimental design was such as to examine the effect of microfilament disruption at specific times in infection in order to determine if microfilaments are utilized at a specific time in the RSV life cycle. The experimental design in the previous study was such as to examine the effect on RSV titers when no microfilament network was present throughout the infectious cycle. In the study by Kallewaard et al., cytochalasin D was added immediately after infection, and left on the cells for the duration of the experiment for 3 days, unlike our study, where the inhibitor was used for a defined (12 h) period. As clearly shown in Figure 2B, removal of cytochalasin D allows the microfilament network to reform, while continued treatment would mean that the microfilament network is disrupted through the whole experimental period.

We have previously shown that the RSV M protein localizes to the nucleus early in infection, maybe to inhibit host transcription [16], while, later in infection, moving out of the nucleus to complete assembly [15,31]. Failure of the M protein to exit the nucleus impairs its accumulation at assembly sites which, in turn, affects viral budding. Nuclear export of M is mediated by Crm-1, and treatment with nuclear export inhibitors has been shown to impair virus production [17]. Here, we show that upon chemical destabilization of the microfilament network, the M protein accumulates in the nucleus correlating with reduced viral titer. These findings suggest that M may require the actin cytoskeleton for movement out of the nucleus. This has been shown for the nuclear–cytoplasmic shuttling of β-dystroglycan (β-DG), which is modulated by cytoskeletal rearrangement [32].

M associates with F-actin directly in vitro, and colocalizes with microfilaments in cell culture. The latter is shown by CLSM and by inference from the fact that chemical destabilization of microfilaments results in mislocalization of M in transfected cells. There was no effect on the localization of the M deletion mutants used in this study. Our data clearly show that the N- or C-terminal of M are not, in themselves, sufficient to mediate M’s interaction with microfilaments in the cellular context. Many proteins that bind to polymerized actin, including those that crosslink microfilaments, form homodimers [33,34,35,36]. Indeed, the dimer form of these proteins is key to their association with microfilaments. M protein also forms dimers and has been crystalized in its dimeric form. Recently, we have shown that RSV M protein can self-interact when expressed in living mammalian cells, and that this requires both the N- and C-terminal domains of the protein, consistent with the reported head to tail dimeric structure of M, whereby the N-terminal domain of one subunit interacts with the C-terminal domain of the other subunit [7,37]*.* Together with the data reported in this study, this suggests that M’s interaction with polymerized actin may require formation of the homodimer; however, this remains to be elucidated.

In conclusion, the microfilament network is required throughout RSV infection, that is, in both early and late infection, for optimal assembly. As the RSV M protein plays a critical role in the transport of the RNP and glycoproteins, and as M’s subcellular localization depends, at least in part on microfilaments, M may interact with F-actin to facilitate movement of these components. Current work in the group is focused on elucidating this aspect of RSV assembly.
